# Multiple T-cell responses are associated with better control of acute HIV-1 infection: An observational study

**DOI:** 10.1097/MD.0000000000004429

**Published:** 2016-07-29

**Authors:** Jianping Sun, Yan Zhao, Yanchun Peng, Zhen Han, Guihai Liu, Ling Qin, Sai Liu, Huanhuan Sun, Hao Wu, Tao Dong, Yonghong Zhang

**Affiliations:** a Beijing You’An Hospital, Capital Medical University, Beijing, PR China; b Medical Research Council Human Immunology Unit, Weatherall Institute of Molecular Medicine, John Radcliffe Hospital, Oxford University, Oxford, UK.

**Keywords:** acute infection, CTL, HIV-1

## Abstract

Cytotoxic T lymphocyte (CTL) responses play pivotal roles in controlling the replication of human immunodeficiency virus type 1 (HIV-1), but the correlation between CTL responses and the progression of HIV-1 infection are controversial on account of HIV immune escape mutations driven by CTL pressure were reported.

The acute HIV-1-infected patients from Beijing were incorporated into our study to investigate the effects of CTL response on the progression of HIV-1 infection.

A longitudinal study was performed on acute HIV-1-infected patients to clarify the kinetic of T-cell responses, the dynamic of escape mutations, as well as the correlation between effective T-cell response and the progression of HIV infection.

Seven human leukocyte antigen-B51+ (HLA-B51+) individuals were screened from 105 acute HIV-1 infectors. The detailed kinetic of HLA-B51-restricted CTL responses was described through blood sampling time points including seroconversion, 3 and 6 months after HIV-1 infection in the 7 HLA-B51+ individuals, by using 16 known HLA-B51 restricted epitopes. Pol743–751 (LPPVVAKEI, LI9), Pol283–289 (TAFTIPSI, TI8), and Gag327–3459 (NANPDCKTI, NI9) were identified as 3 dominant epitopes, and ranked as starting with LI9, followed by TI8 and NI9 in the ability to induce T-cell responses. The dynamics of escape mutations in the 3 epitopes were also found with the same order as T-cell response, by using sequencing for viral clones on blood sampling at seroconversion, 3 and 6 months after HIV-1 infection.

We use solid evidence to demonstrate the correlation between T-cell response and HIV-1 mutation, and postulate that multiple T-cell responses might benefit the control of HIV-1 infection, especially in acute infection phase.

## 1 Introduction

It was well accepted that cluster of differentiation 8^+^ (CD8^+^) T cells play a pivotal role in controlling human immunodeficiency virus type 1 (HIV-1) replication. For one thing, a temporal association is observed between the rise of virus-specific cytotoxic T lymphocyte (CTL) responses and the reduction of viremia during acute phase of HIV-1 infection, beyond that, the peak of the CTL response coincides with the drop in viral load.^[1,2]^ For another, in the nonhuman primate models with simian immunodeficiency virus (SIV) infection, the depletion of CD8^+^ T cells during the acute phase of SIV infection,^[3]^ or chronic SIV infection results in a rapid increase of viral load that is again suppressed when the SIV-specific CD8^+^ T cells reappear.^[4]^

Furthermore, the escape mutations detected in the CTL epitopes reveal the evidence of immune pressure is exerted by CTL responses.^[5]^ The first escape variants can replace the original sequence of the founder virus within days, and as a consequence of continuous immune pressure, sequential mutations at different epitopes are followed.^[6]^ Eventually, the frequency of escape mutations correlates with the prevalence of the restricting human leukocyte antigen (HLA) class I allele at the population level.^[5,7,8]^ While the correlation between CTL responses and the progression of HIV-1 infection became controversial, because of viral escape is also driven by CTL pressure, which is the key roadblock to the successful development of an efficient T-cell-based HIV-1 vaccine.^[9]^

Several strategies are employed to design an effective T-cell-based HIV-1 vaccine, from immunodominant^[10]^ and subdominant epitopes^[11]^ to conserved^[12]^ or mosaic^[13]^ epitopes. Amongst these strategies, the kinetics and quality of early immune responses to HIV-1 are not very clear, while their implications for developing a successful preventive HIV-1 vaccine have become more and more valuable.

HLA-B51 is shown to be one of the protective class I alleles associated with delayed disease progression of HIV infection, especially in Asian population.^[7]^ HLA-B51 restricted epitopes including Gag327–345 (NANPDCKTI, NI9), Pol743–751 (LPPVVAKEI, LI9), and Pol283–289 (TAFTIPSI, TI8), as well as mutations in these 3 dominant epitopes are identified in our previous study.^[14]^ The kinetics of T-cell responses and the dynamic of escape mutations during acute phase of HIV-1 infection were demonstrated in this study to clarify the correlation between the T-cell responses, escape mutations, and disease progression.

We selected 7 HLA-B51+ individuals from acute HIV-1 infection cohort, and the kinetic of HLA-B51-restricted CTL responses was described through time points of seroconversion, 3 and 6 months after HIV-1 infection, by using 16 known HLA-B51-restricted epitopes from the Los Alamos Molecular Immunology database. Three epitopes—Pol743–751 (LI9), Pol283–289 (TI8), and Gag327–345 (NI9)—were ranked as starting with LI9 responses, followed by TI8 and NI9. The dynamics of escape mutations in these 3 epitopes were also found by using sequencing for viral clones, which were extracted and amplified from the same individuals on time points of seroconversion, 3 and 6 months of HIV-1 infection. We use solid evidence to demonstrate the correlation between T-cell response and viral mutation, and postulate that multiple effective T-cell responses (ETRs) might benefit the control of HIV-1 infection, especially in acute infection phase.

## 2 Materials and methods

### 2.1 Study population

A total of 6000 candidates from a homosexual cohort study of primary HIV-1-infected individuals in Beijing were monitored every 2 months for plasma HIV antibodies, HIV ribonucleic acid (RNA) levels, and clinical signs of acute infection to screen acute HIV-1-infected patients as we described in the previous study.^[15]^ Amongst them, 105 candidates were defined as acute HIV-1-infected patients and recruited in this study. Whole-blood specimens were collected every 3 months, thereafter from detection of seroconversion and used to separate plasma, peripheral blood mononuclear cells (PBMCs), and genomic deoxyribonucleic acid. Among these HLA-B51-positive individuals, patients with less than 1 ETR were segregated into Group 1, and Group 2 were patients with at least 2 ETRs.

### 2.2 Clinical definitions and laboratory detection

Acute HIV-1 infection was defined as the positive testing of HIV-1 RNA and negative or indeterminate testing by enzyme-linked immunosorbent assay (ELISA) and Western blot of HIV-1.^[16]^ For detection of specific antibodies to HIV-1, standard HIV-1 ELISA (Abbott, Abbott Park, IL) and Western blot analysis (Genelabs, Redwood City, CA) kits were used. The copies of HIV-1 RNA were quantified in serum with Roche Amplicor kit (Roche Molecular Diagnostics, Indianapolis, IN). The absolute counts of CD3^+^/CD4^+^/CD8^−^ T cells (CD4^+^T) and CD3^+^/CD4^−^CD8^+^ T cells (CD8^+^T) were measured by using TriTEST Three-Color reagents (BD Company, FranklinLakes, NJ) and MultiSET software in the FACSCalibur Flow Cytometry System (BD Company, FranklinLakes, NJ).

### 2.3 HLA genotyping

Low-resolution (2-digit) HLA class I molecular typing was performed with sequence-based typing at SinoGenoMax, Chinese National Human Genome Center of Beijing, Beijing, PR China. HLA graphing was performed by HLA analysis tools from HIV molecular immunology database (http://www.hiv.lanl.gov/content/immunology/hla/hla_graph.html).

### 2.4 HIV-1 clone and sequencing

HIV-1 RNAs were extracted from plasma on time points of seroconversion, 3 and 6 months after HIV-1 infection, and then were cloned into pCR™4-TOPO^®^ TA vector through the TA TOPO cloning kit (Invitrogen, Carlsbad, CA). Expected 20 colonies were constructed from each sample, and HIV-1 *gag* and *pol* genes were amplified and sequenced by Beijing Institute of Genomics, Shenzhen, China.

### 2.5 Human IFN-gamma ELISPOT assay

A total of 200,000 PBMCs with 10 μg/mL peptide were used in a standard human interferon (IFN)-gamma enzyme-linked immunospot assay (ELISPOT) assays as described before.^[14]^ Briefly, assays were carried out in 96-well MultiScreen filter plates (Millipore, MAIP S45, Millipore, MA) coated with 15 mg/mL anti-IFN-gamma monoclonal antibody (1-DIK; Mabtech, Sweden). A total of 5 μg/mL phytohemagglutinin (final concentration, 1 μg/mL) and RPMI1640 were used as positive and negative control, respectively. Plates were incubated for 16 hours at 37°C, 5% CO_2_. Spot enumeration was performed with ELISPOT ImmunoSpot Analyzers (Cellular Technology Limited, East Asia, Beijing, China). To quantify HIV-specific responses, mean spots of negative control were subtracted from the positive wells, and results were expressed as spot-forming units (SFUs) per 10^6^ PBMCs. Responses were regarded as positive if results were at least 3 times as the mean of the negative control wells and above 50 SFUs/10^6^ PBMCs.

### 2.6 Definition of effective T-cell response

According to our previous study,^[14]^ epitopes Pol743–751 (LI9), Pol283–289 (TI8), and Gag327–345 (NI9) were confirmed as the protection roles in the control of HIV replication, and their mutations escapes were driven by CTL pressure. Hence, ETR was defined as a T-cell response activated by 1 epitope and without mutation at the same time.

### 2.7 Ethics

The study was approved by the Institutional Review Board of Beijing You’An Hospital. The ethics committee approved the relating screening, inspection, and data collection of the patients, and all subjects signed a written informed consent form. All works were undertaken following the provisions of the Declaration of Helsinki.

### 2.8 Statistical analysis

Statistical analysis of the genetic data was performed using nonparametric *t* test (Mann–Whitney test). *P* value less than 0.05 was considered statistical difference. Analyses were performed with the GraphPad Prism version 5.0 software (La Jolla, CA).

## 3 Results

### 3.1 Clinical and laboratory characteristics of study participants

Amongst 105 acute HIV-1-infected individuals, 7 were identified as HLA-B51 positive, and 98 candidates were identified as HLA-B51 negative; the population frequency of HLA-B51 in this cohort was 6.73%. No significant difference in cluster of differentiation 4^+^ (CD4^+^) T cells count between HLA-B51-positive and HLA-B51-negative individuals was observed at seroconversion (569.28 ± 268.23 vs 505.82 ± 177.00 cells/μL; *P* = 0.636), 3 months (472.57 ± 214.52 vs 512.05 ± 190.75 cells/μL; *P* = 0.435), and 6 months (506.00 ± 384.79 vs 479.18 ± 199.75 cells/μL; *P* = 0.754) after HIV infection. In addition, no significant difference was observed in HIV-1 viral load between HLA-B51-positive and HLA-B51-negative individuals (Table 1).

**Table 1:**
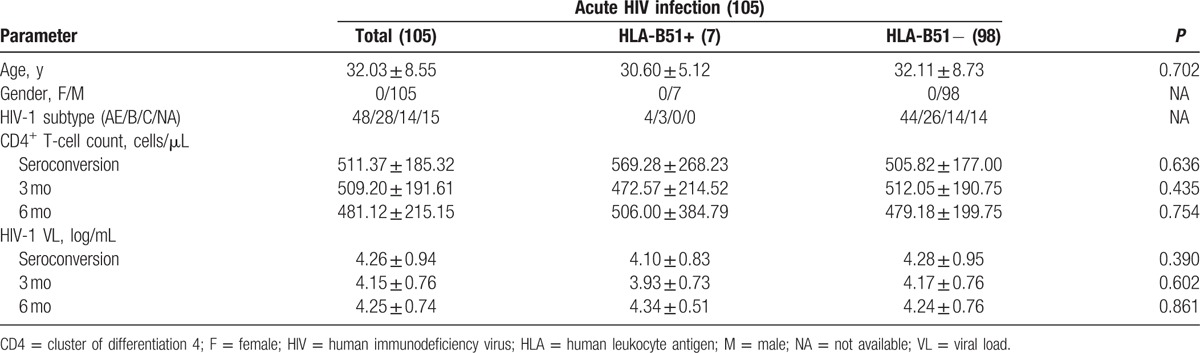
Clinical and laboratory characteristics.

### 3.2 Kinetic of dominant HLA-B51-restricted HIV-1 specific T-cell epitopes in acute HIV-1 infection cohort

**Figure 1:**
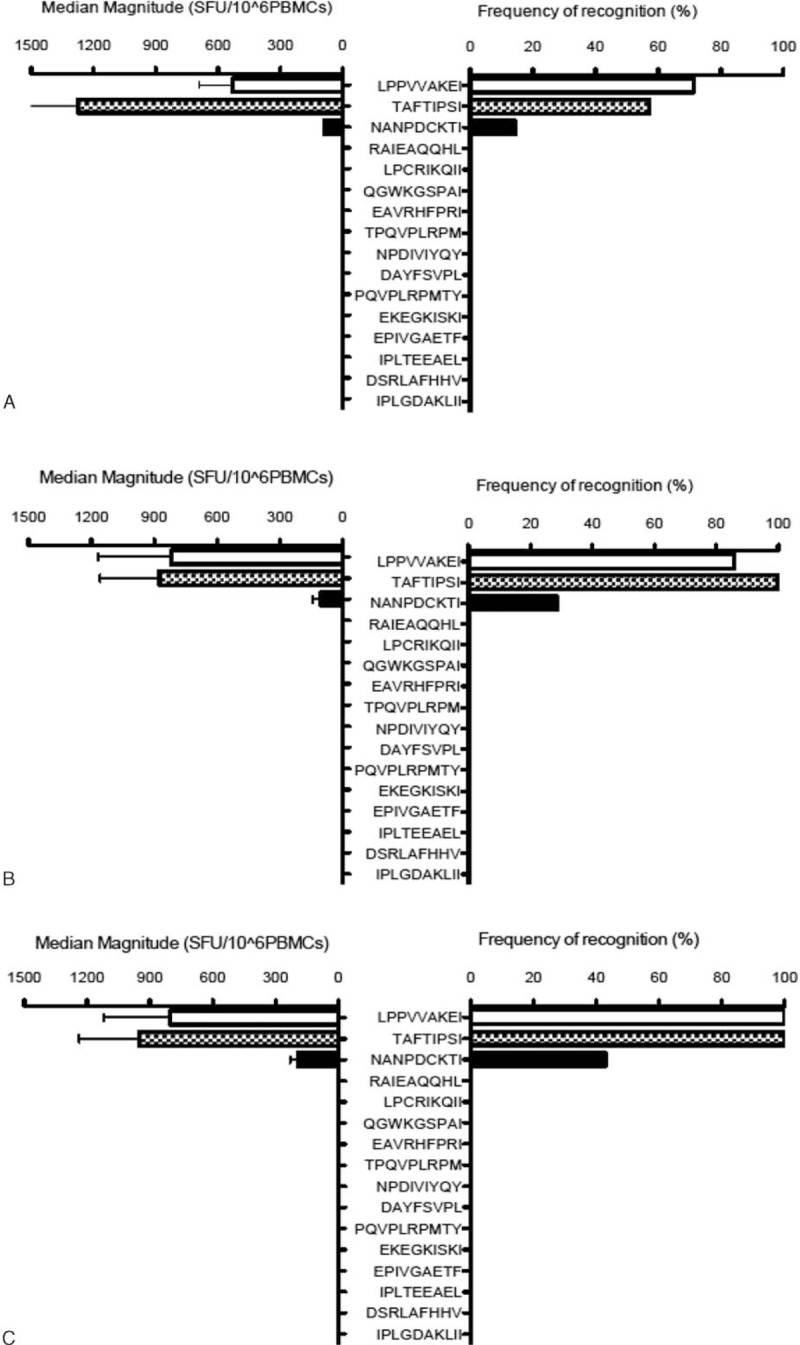
The magnitude of response and frequency of recognition in human leukocyte antigen-B51 positive human immunodeficiency virus type 1 infected patients at seroconversion (A), 3 months (B), and 6 months (C).

**Figure 2:**
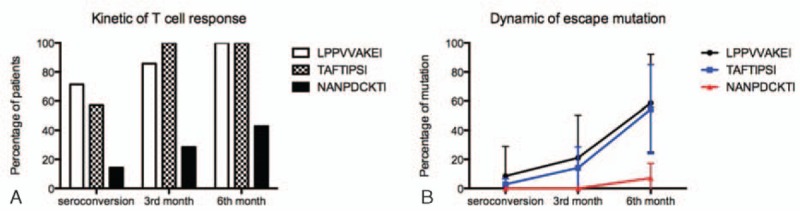
The kinetic of T-cell response and dynamic of escape mutation. The frequency of recognition (A) and the percentage of mutations (B) of immunodominant epitopes, including Gag327–345 (NI9), Pol743–751 (LI9), and Pol283–289 (TI8), in human leukocyte antigen-B51 positive human immunodeficiency virus type 1 (HIV-1)-infected patients at seroconversion, 3 and 6 months after acute HIV-1 infection.

The kinetic of HLA-B51-restricted CTL responses was described through time points of seroconversion, 3 and 6 months of HIV-1 infection, by using 16 known HLA-B51-restricted epitopes in 7 HLA-B51+ individuals from acute HIV-1 infection cohort. Epitopes including Pol743–751 (LI9), Pol283–289 (TI8), and Gag327–345 (NI9) were identified as dominant epitopes, and other known HLA-B51-restricted epitopes had no response (Fig. 1). Five out of 7 (71.4%) HLA-B51-positive HIV-1-infected individuals responded to epitope LI9 at seroconversion, and then the percentage of T-cell response increased to 85.7% (6/7) at 3 months and 100% (7/7) at 6 months of HIV-1 infection. The response of epitope TI8 was 57.1% of HLA-B51-positive HIV-infected individuals at seroconverstion, increased to 100% at 3 and 6 months of HIV-1 infection. Epitope NI9 response changed from 14.3% at seroconversion to 28.6% on 3 months and 42.8% on 6 months of HIV-1 infection (Fig. 2A).

### 3.3 Dynamic of escape mutations driven by dominant epitopes

Based on our previous study, we found that the escape mutations of epitopes—Pol743–751 (LI9), Pol283–289 (TI8), and Gag327–345 (NI9)—were associated with HLA-B51-specific CTL response.^[14]^ Then we identified the dynamics of escape mutations in these 3 epitopes by using sequencing for viral clones, which extracted and amplified from the same individuals at seroconversion, 3 and 6 months of HIV-1 infection. As shown in Table 2 and Fig. 2B, epitopes LI9 mutated first at seroconversion, the mutation rate was from 8.57%, increased to 21.06% at the 3 months, and to 58.57% at the 6 months of HIV-1 infection; epitope TI8 escape mutation rate was found from 2.86% at seroconversion, increased to 14.13% at 3 months and 54.49% at the 6 months of HIV-1 infection. Epitope NI9 mutated later than LI9 and TI8, only 7.13% of variations were identified at 6 months of acute HIV-1 infection.

**Table 2:**
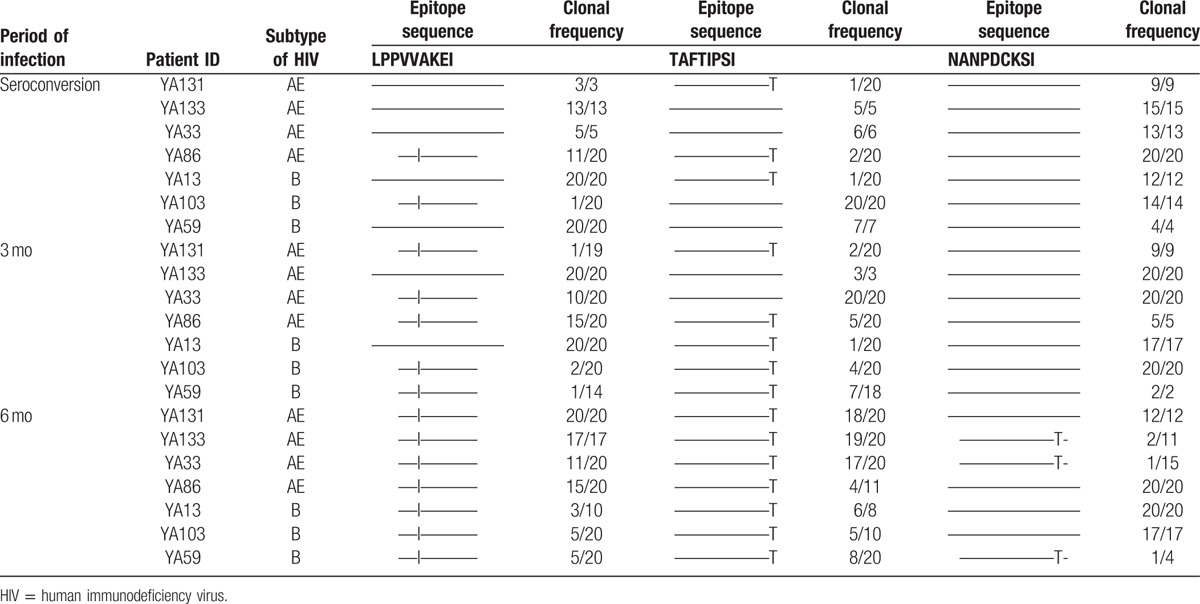
The rate of mutations in epitope LI9, TI8, and NI9 at seroconversion, 3 and 6 months of acute human immunodeficiency virus type 1 infection.

### 3.4 Correlation between multiple effective T-cell responses and disease progression

**Figure 3:**
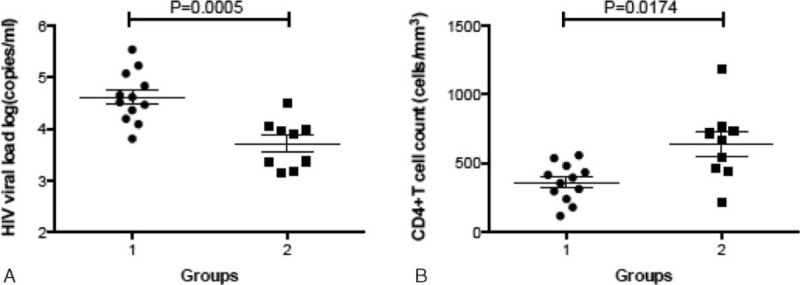
Multiple effective T-cell responses (ETRs) were strongly associated with better clinical outcome. The human immunodeficiency virus type 1 viral load (A) and cluster of differentiation 4^+^ T-cell counts (B) were compared between Group 1 (patients with less than 1 ETR) and Group 2 (patients with at least 2 ETRs).

**Figure 4:**
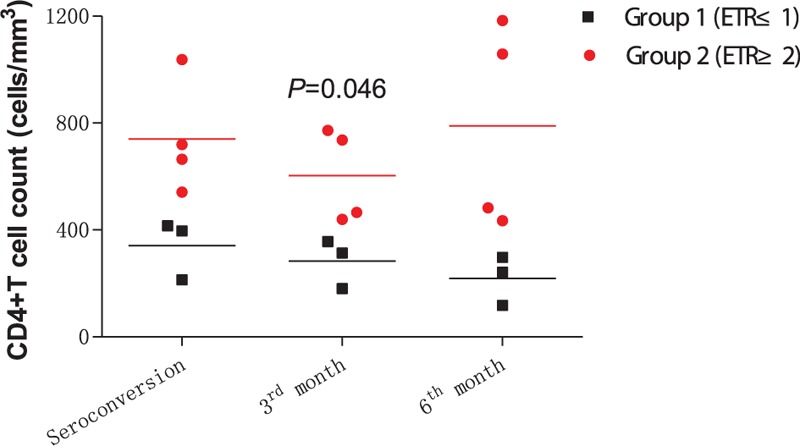
The difference of dynamic of cluster of differentiation 4^+^ T-cell counts based on patients with various effective T-cell response (ETR) at seroconversion, 3 and 6 months after human immunodeficiency virus type 1 infection. Patients in Group 1 with less than 1 ETR were identified as the black square, and patients in Group 2 with at least 2 ETRs were presented with red circle.

We evaluated the status of ETR at seroconversion, the third month, and the sixth month after HIV infection according to corresponding T-cell response and viral mutation. And then, the correlation between ETR status and the disease progression was performed by analyzing the differences between various ETR in CD4 counts and plasma viral load. A significant difference was found in HIV viral load between Group 1 (ETR ≤ 1, 4.616 ± 0.143) and Group 2 (ETR ≥ 2, 3.715 ± 0.156, *P* = 0.0005, Fig. 3A). Moreover, CD4^+^ T-cell count in Group 2 (636.9 ± 90.63 cells/μL) was much higher than that in Group 1 (360.5 ± 39.6 cells/μL, *P* = 0.0174, Fig. 3B). In addition, the change of CD4 cell counts was further confirmed from seroconversion to 6 months after HIV-1 infection. At 3 months, the CD4 cell counts of patients in Group 2 (ETR ≥ 2) was significantly higher than those patients in Group 1 (ETR ≤ 1) (*P* = 0.0046, Fig. 4).

## 4 Discussion

It has been demonstrated that HLA-B51 is one of the protective class I alleles associated with delayed disease progression of HIV infection.^[7,14]^ We previously found that the HLA-B51-restricted epitopes, Gag327–345 (NANPDCKTI, NI9), Pol743–751 (LPPVVAKEI, LI9), and Pol283–289 (TAFTIPSI, TI8), as well as mutations in these 3 dominant epitopes were associated with the progress of HIV disease.^[14]^ Here, the time points of acute phase of HIV-1 infection including seroconversion, 3 and 6 months of HIV infection were provided to describe the kinetics of T-cell responses and the dynamic of escape mutations.

In this study, we described the detailed kinetic of dominant epitopes restricted by HLA-B51 during the first half year of acute HIV-1 infection. Epitope Pol743–751 (LI9) and epitope Pol283–289 (TI8) were first identified at seroconversion of acute HIV-1 infection by 71.4% (to LI9) and 57.1% (to TI8) in HLA-B51-positive population, then the frequencies of recognition were increased to 100% of HLA-B51-positive HIV-1-infected patients at the end of 6 months of HIV-1 infection. Moreover, the change of T-cell response stimulated by epitope Gag327–345 (NI9) was found from rare at the seroconversion to common at 6 months of HIV infection. This comprehensive longitudinal T-cell responses profile provided us research foundation to further discuss the role of T-cell responses in the control of HIV-1 progression.

We also employed clone sequencing to reveal the dynamic of escape mutations in dominant epitopes. Interestingly, the same rank of change was found comparing with T-cell response. Epitopes LI9 and TI8 mutated first at seroconversion, and the mutation rate was from less than 10% to over 50% at 6 months of HIV-1 infection; and epitope NI9 mutated quite lately, only 7.13% of variations were identified until at 6 months of acute HIV-1 infection. This longitudinal data not only solidly supported CTL pressure had a major effect on interhost HIV-1 viral diversity,^[5]^ but also confirmed our previous proposal in chronic HIV-1 infection cohort study^[14]^ in which HLA-B51-restricted epitopes mutated regularly, epitope LI9 mutated first, followed by epitope TI8, and then epitope NI9.

In our previous study, we found that the viral load of HLA-B51-positive individuals is lower than those of HLA-B51-negative individuals.^[14]^ But here, the viral load of HLA-B51-positive individuals was not significant with those of HLA-B51-negative individuals, which might be caused by the HLA-B51-positive individuals being only 7. In here, the most important finding of this study was that we integrated T-cell response and escape mutations together to evaluate the ETR, which was correlated with the disease progression. A clear phenomenon was found that those patients with more than 2 ETRs had the lower HIV viral load and the higher CD4^+^ T-cell count, comparing with those with less than 1 ETR. This finding strongly exposed the importance of multiple effective T cell to the control of progression in acute HIV-1 infection, which could be used as a new strategy in the selection of T-cell vaccine to HIV infection.

In conclusion, our data explicitly showed that the kinetic of dominant epitopes restricted by HLA-B51 during the first half-year of acute HIV-1 infection, the dynamic of escape mutations driven by dominant epitopes, and the correlation between multiple ETRs and disease progression. This finding not only confirmed our previous study about the majority of viral mutation under CTL driving pressure, but also expanded the role of ETR to control the disease progression.
